# Mysterious Influences

**Published:** 1872-09

**Authors:** 


### MYSTERIOUS INFLUENCES.

PERSONS sometimes feel remarkably well, — the appetite is vigorous, eating is a joy, digestion vigorous, sleep sound, with an alacrity of body and an exhilaration of spirits, which altogether throw a charm over life that makes us pleased with everybody and everything. Next week, to-morrow, in an hour, a marvelous change comes over the spirit of the dream; the sunshine has gone, clouds portend, darkness covers the face of the great deep, and the whole man, body and soul, wilts away like a flower without water in midsummer.

A bee has wandered far away from its hive on a beautiful June morning; every flower is unfolded, ready to yield all its treasures of sweetness; suddenly he speeds away towards his home with an arrow's swiftness, for a cloud has come between him and the sun, and forebodings come of ill; his little heart is just as full of hurried hasting for home, as but a moment before it was of hope to get its fill of honey.

Both bee and man are affected by changes in the condition of the atmosphere; many fly before an east wind, as a bee before a cloud, the electrical conditions of the atmosphere having been changed in both cases.

When the weather is cool and clear and bracing, the atmosphere is full of electricity; when it is sultry and moist, and without sunshine, it holds but a small amount of electricity, comparatively speaking, and we have to give up what little we have, moisture being a good conductor; thus, in giving up, instead of receiving more, as we would from the cool, pure air, the change is too great, and the whole man languishes. Many become uneasy under these circumstances; "they can't account for it;" they imagine that evil is impending, and resort at once to tonics and stimulants. The tonics only increase the appetite, without imparting any additional power to work up the additional food, thus giving the system more work to do, instead of less. Stimulants seem to give more strength; they wake up the circulation, but it is only temporarily, and unless a new supply is soon taken, the system runs further down than it would have done without the stimulant; hence it is in a worse condition than if none had been taken. The better course would be to rest, take nothing but cooling fruits and berries and melons, and some acid drink, when thirsty, adding, if desired, some cold bread and butter; the very next morning will bring a welcome change.

